# SPON1 is an independent prognostic biomarker for ovarian cancer

**DOI:** 10.1186/s13048-023-01180-8

**Published:** 2023-05-13

**Authors:** Ryoya Miyakawa, Makoto Kobayashi, Kotaro Sugimoto, Yuta Endo, Manabu Kojima, Yasuyuki Kobayashi, Shigenori Furukawa, Tsuyoshi Honda, Takafumi Watanabe, Shigeyuki Asano, Shu Soeda, Yuko Hashimoto, Keiya Fujimori, Hideki Chiba

**Affiliations:** 1 Department of Basic Pathology, Fukushima Medical University School of Medicine, Fukushima, 960- 1295 Japan; 2 Department of Obstetrics and Gynecology, Fukushima Medical University School of Medicine, Fukushima, 960-1295 Japan; 3 Department of Regional Gynecologic Oncology, Fukushima Medical University School of Medicine, Fukushima, 960-1295 Japan; 4 Department of Diagnostic Pathology, Fukushima Medical University School of Medicine, Fukushima, 960-1295 Japan; 5 Department of Regional Medical Support for Obstetrics and Gynecology, Fukushima Medical University School of Medicine, Fukushima, 960-1295 Japan; 6 Department of Obstetrics and Gynecology, Iwaki City Medical Center, Iwaki, 973-8555 Japan; 7 Department of Pathology, Iwaki City Medical Center, Iwaki, 973-8555 Japan

**Keywords:** F-spondin, Gynecological cancer, Prognosis, Recurrence, Spondin-1

## Abstract

**Background:**

Ovarian cancer has the worst outcome among gynecological malignancies; therefore, biomarkers that could contribute to the early diagnosis and/or prognosis prediction are urgently required. In the present study, we focused on the secreted protein spondin-1 (SPON1) and clarified the prognostic relevance in ovarian cancer.

**Methods:**

We developed a monoclonal antibody (mAb) that selectively recognizes SPON1. Using this specific mAb, we determined the expression of SPON1 protein in the normal ovary, serous tubal intraepithelial carcinoma (STIC), and ovarian cancer tissues, as well as in various normal adult tissues by immunohistochemistry, and verified its clinicopathological significance in ovarian cancer.

**Results:**

The normal ovarian tissue was barely positive for SPON1, and no immunoreactive signals were detected in other healthy tissues examined, which was in good agreement with data obtained from gene expression databases. By contrast, upon semi-quantification, 22 of 242 ovarian cancer cases (9.1%) exhibited high SPON1 expression, whereas 64 (26.4%), 87 (36.0%), and 69 (28.5%) cases, which were designated as SPON1-low, possessed the moderate, weak, and negative SPON1 expression, respectively. The STIC tissues also possessed SPON1-positive signals. The 5-year recurrence-free survival (RFS) rate in the SPON1-high group (13.6%) was significantly lower than that in the SPON1-low group (51.2%). In addition, high SPON1 expression was significantly associated with several clinicopathological variables. Multivariable analysis revealed that high SPON1 was an independent prognostic factor for RFS of ovarian cancer.

**Conclusions:**

SPON1 represents a prognostic biomarker for ovarian cancer, and the anti-SPON1 mAb could be valuable as an outcome predictor.

**Supplementary Information:**

The online version contains supplementary material available at 10.1186/s13048-023-01180-8.

## Background

Ovarian cancer is the fifth-leading cause of female cancer deaths, and it is estimated that 150,000 women with ovarian cancer die annually worldwide [1–3]. Among histological subtypes of epithelial ovarian cancer (EOC), high-grade serous ovarian cancer is the most common type, and others include low-grade serous, endometrioid, clear cell, and mucinous ovarian cancers [4–9]. Approximately 75% of EOC patients are diagnosed at the advanced stages because of the asymptomatic nature of EOC and the lack of an effective screening tool [8]. In addition, even though most EOC patients achieve complete remission by surgery and cisplatin-based chemotherapy, the recurrence-free survival (RFS) rate after two years remains around 25% [10]. Furthermore, relapsed EOC is basically incurable. Given these sorts of difficulties, prognosis of ovarian cancer is enormously poor compared with other gynecological malignancies, and the 5-year overall survival rate is only 45% [11]. Therefore, there is an urgent need for novel biomarkers that are able to predict the EOC outcome at the time of initial surgery, as well as to diagnose EOC as early as possible.

Spondin-1 (SPON1; F-spondin; vascular smooth muscle cell growth-promoting factor) is a secreted protein that belongs to the thrombospondin family. It consists of an N-terminal reelin domain, the spondin domain, and thrombospondin type I repeats. SPON1 was originally identified by subtractive hybridization as a factor that induces neural cell adhesion and axon guidance in the vertebrates [12]. SPON1 also contributes to neuronal differentiation [13] and regulation of circadian rhythms [14]. In addition, it was reported that SPON1 binds to amyloid-β precursor protein (APP), which plays important roles in the development and progression of Alzheimer’s disease [15]. Interestingly, Pyle-Chenault et al. identified that *SPON1* gene expression is significantly upregulated in ovarian cancer tissues by cDNA library subtractions [16]. Nevertheless, the clinicopathological relevance of SPON1 protein expression in ovarian cancer has not been clarified so far, likely due to the absence of selective antibodies.

In the present study, we developed a monoclonal antibody (mAb) that specifically reacts with human SPON1 and works for immunohistochemical staining using formalin-fixed paraffin-embedded (FFPE) tissues. Using this SPON1 mAb, we demonstrated that the RFS in the SPON1-high group of ovarian cancer subjects is significantly lower than that in the SPON1-low group, and that high SPON1 expression is associated with various clinicopathological factors. Furthermore, we also showed that high SPON1 is an independent prognostic marker for ovarian cancer.

## Materials and methods

### Generation of antibodies

Rat mAbs against SPON1 were established using the iliac lymph node method [17]. In brief, a polypeptide, (C)EKTHPKDYPRRANHWSAI, corresponding amino acid number 217–234 of human SPON1, was coupled via the cysteine to Imject Maleimide-Activated mcKLH (Thermo Fisher Scientific, Waltham, MA, USA). The conjugated peptide was intracutaneously injected with Imject Freund’s Complete Adjuvant (Thermo Fisher Scientific) into the footpads of anesthetized eight-week-old female rats. All animal experiments complied with the National Institutes of Health Guide for the Care and Use of Laboratory Animals, and were approved by the Animal Committee of Fukushima Medical University (FMU) (approval code, 2021-092; approval date, 10 May 2021). The animals were sacrificed 14 days after immunization, and the median iliac lymph nodes were collected, followed by extraction of lymphocytes by mincing. The extracted lymphocytes were fused with cells of the SP2 mouse myeloma cell line using polyethylene glycol. Hybridoma clones were maintained in GIT medium (Wako, Osaka, Japan) with supplementation of 10% BM-Condimed (Merck Millipore, Burlington, MA, USA). The supernatants were screened by enzyme-linked immunosorbent assay (ELISA).

### Cell culture, expression vectors and transfection

The human ovarian cancer cell lines AMOC2, OVCAR3, and SKOV3, as well as 293T cells were grown in Dulbecco’s Modified Eagle Medium (DMEM, Merck Millipore) with 10% fetal bovine serum (FBS; Merck Millipore) and 1% penicillin-streptomycin mixture (Merck Millipore). The protein-coding regions of human SPON1 were cloned into the *XhoI*/*HindIII* site of the pcDNA3.1/Hygro(+) plasmid (Addgene, Watertown, MA, USA). For transient expression of the above-mentioned target genes, 5 × 10^6^ 293T cells were transfected with 10 µg of the indicated vectors using 30 µg of Polyethylenimine Max (#24765-1; Polysciences, PA, USA) 8 h after passage.

### Immunoblotting

Total cell lysates were collected with CelLytic MT Cell Lysis Reagent (Merck Millipore), followed by one-dimensional SDS-PAGE, and were electrophoretically transferred onto a piece of Immobilon (Millipore, Burlington, MA, USA). The membrane was saturated with phosphate-buffered saline (PBS) containing 4% skimmed milk (Morinaga) and treated with primary antibodies. Supernatants of rat anti-SPON1 hybridoma were directly used as primary antibodies. The signal was detected by chemiluminescence using 2,000-fold diluted horseradish peroxidase (HRP)-conjugated anti-rat IgG (NA935V, GE Health Care, Chicago, IL, USA).

### Cell blocks

Cells were centrifuged at 1,200 rpm for 10 min and fixed with 10% formalin for 16 h at 4 °C. Fixed cell pellets were mixed with 1% sodium alginate followed by 1 M calcium chloride and embedded in paraffin using Tissue-Tek VIP 5 Jr (Sakura Finetek Japan, Tokyo, Japan).

### Isolation of ovarian cancer cells from ascites fluid

Five-hundred mL of ascites fluid was obtained from a 52-year-old female ovarian cancer patient. The ascites fluid was centrifuged at 2,000 rpm for 15 min and precipitated cells were washed three times with red blood cell lysis buffer (1 mM ammonium bicarbonate and 114 mM ammonium chloride). Cell pellets were fixed with 10% formalin for 16 h at 4 °C to prepare the cell block using the same methods described above.

### ELISA

The antigen peptide was adsorbed onto Nunc-Immuno MaxiSorp plates (44-2404-21; Thermo Fisher Scientific) overnight at 4ºC. After washing with PBS, non-specific reactivity was blocked by 1% bovine serum albumin (BSA)/PBS for 30 min. The wells were incubated with the hybridoma supernatant for 1 h at 37ºC as the primary antibody. After washing with PBS, the plate was incubated with 2,000-fold diluted HRP-conjugated goat anti-rat antibody (NA935; Cytiva, Tokyo, Japan) for 1 h at 37ºC. 3,3’,5,5’ tetramethyl benzidine substrate kit (421,101, Biolegend, San Diego, CA) was used for the detection.

### Tissue collection

FFPE tissue sections were obtained from: 167 patients with ovarian cancer (age, 18–87 years; average ± SD = 57.3 ± 13.1) who underwent hysterectomy alone or together with bilateral salpingooophorectomy and/or lymphadenoectomy between 2003 and 2015 at FMU Hospital; and 75 patients with ovarian cancer (age, 25–83 years; average ± SD = 54.7 ± 11.4) who underwent the same operation described above between 2010 and 2015 at Iwaki City Medical Center. The ovarian cancer subjects were limited to patients who were confirmed to have at least 5-year outcomes and those who had died due to ovarian cancer and metastasis. Detailed information, including postoperative pathology diagnosis reports, age, histological type, stage (International Federation of Gynecology and Obstetrics [FIGO] 2008), ascites cytology, peritoneal dissemination, lymph node metastasis, distant metastasis, chemotherapy, intent of surgery, serum Cancer antigen-125 (CA-125) concentration, recurrence status, disease-specific survival (DSS), and RFS, were obtained. Distant metastasis was judged by diagnostic imaging. Four specimens of STIC were collected at FMU Hospital in 2021, and normal adult healthy brain, heart, liver, kidney, lung, pancreas, testis, and thyroid gland, and uterus tissues were obtained from six autopsy cases dissected at FMU Hospital between January 2018 and December 2019. The study was approved by the Ethics Committee of FMU (approval code, 2019 − 311; approval date, 18 March 2020; and approval code, 2021-057; approval date, 16 March, 2021).

### Immunostaining and analysis

FFPE tissue blocks were sliced into 5-µm-thick sections, deparaffinized with xylene, and rehydrated using a graduated series of ethanol. The sections were then immersed in 0.3% hydrogen peroxide in methanol for 20 min at room temperature (RT) to block endogenous peroxidase activity. For α-smooth muscle actin (αSMA), D2-40, and cytokeratin, antigen retrieval was performed by incubating the sections in boiling 10 mM citric acid buffer (pH 6.0) in a microwave. After cooling at RT for 30 min, the sections were blocked with 5% skimmed milk for 30 min. After blocking, the sections were incubated overnight at 4ºC with 100-fold diluted anti-αSMA (M0851; Agilent Technologies, Santa Clara, CA, USA), 50-fold diluted anti-D2-40 (M3619; Agilent Technologies) and anti-cytokeratin (IR053; Agilent Technologies) as the primary antibodies. After washing with PBS, a secondary antibody reaction was performed by using the Histofine Simple Stain mouse MAX‑PO kit (424151; Nichirei Biosciences, Tokyo, Japan) for 3’,3’-diaminobenzidine (DAB) (Stable DAB; Thermo Fisher Scientific) as a chromogen according to the manufacturer’s instructions. For SPON1, antigen retrieval was performed by incubating the sections in boiling Tris-EDTA buffer pH 9.0 (10 mM Tris and 1 mM EDTA with 0.05% tween20) using a microwave. Endogenous avidin and biotin were blocked by an avidin/biotin blocking kit (Nichirei, Tokyo, Japan) at RT for 10 min, and non-specific antibody binding was prevented by 0.5% casein (Merck Millipore) at RT for 10 min. They were then incubated overnight at 4 °C with supernatants of the rat anti-SPON1 hybridoma (clone #1). After washing with TBS2T (11.5 mM Tris-base, 38 mM Tris-hydrocholoride, 300 mM Sodium chloride with 0.1% Tween20), a secondary antibody reaction was performed by using the Histofine mouse PO-Rat secondary antibody (414311; Nichirei, Tokyo, Japan) for 15 min at RT. Next, to amplify signal intensity, sections were serially incubated with 1.5 nM biotinyl tyramide (Merck Millipore) for 15 min at RT and 500-fold diluted HRP-conjugated Streptavidin (Agilent technologies, Santa Clara, CA, USA) for 15 min at RT with DAB as a chromogen according to the manufacturer’s instructions.

Immunostaining results were interpreted by two independent pathologists (K.S. and Y.K.) and a gynecologist (Y.E.) using a semi-quantitative scoring system (immunoreactive score; [IRS [18]]). The immunostaining reactions were evaluated according to signal intensity (SI: 0, no stain; 1, weak; 2, moderate; 3, strong) and percentage of positive cells (PP: 0, < 1%; 1, 1–10%; 2, 11–30%; 3, 31–50%; and 4, > 50%). The SI and PP were then multiplied to generate the IRS for each case, and average IRS score was calculated among three-evaluators. To determine the optical cut-off values of IRS for SPON1 expression, the receiver operating characteristic (ROC) curve was plotted and analyzed. Based on this analysis, we divided the samples into two groups: SPON1-low (average IRS < 10) and SPON1-high (average IRS ≥ 10).

**Fig. 1:**
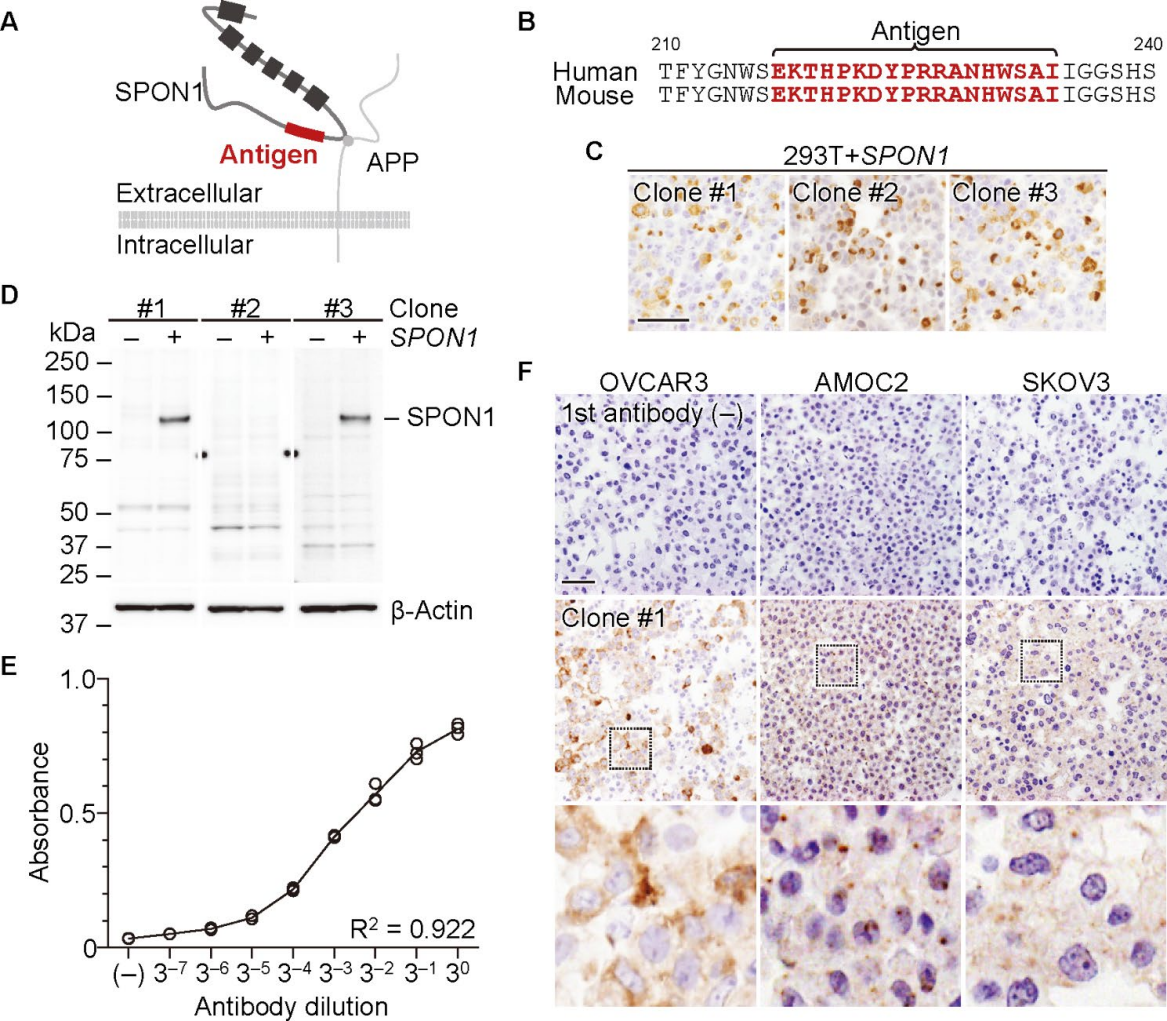
**Establishment and characterization of a rat anti-human SPON1 monoclonal antibody (mAb).** (A) Topology of SPON1. The squares and circle indicate the thrombospondin domain of SPON1 and the binding region of APP (amyloid-β precursor protein) to SPON1. (B) Amino acid sequences of the N-terminal domains of human and mouse SPON1. The antigen region is highlighted in red. Immunohistochemical (C) and Western blot (D) analyses showing the specificity of the indicated mAbs. 293T cells transfected with SPON1 and empty vectors were subjected to these analyses. Scale bar, 100 μm. (E) ELISA analysis revealing the dose-dependent response of the anti-SPON1 mAb (clone #1) to the SPON1 polypeptide. (F) Immunohistochemical images of SPON1 in the indicated ovarian cancer cell lines. Cell slices were reacted with or without clone #1. Scale bar, 100 μm

**Fig. 2:**
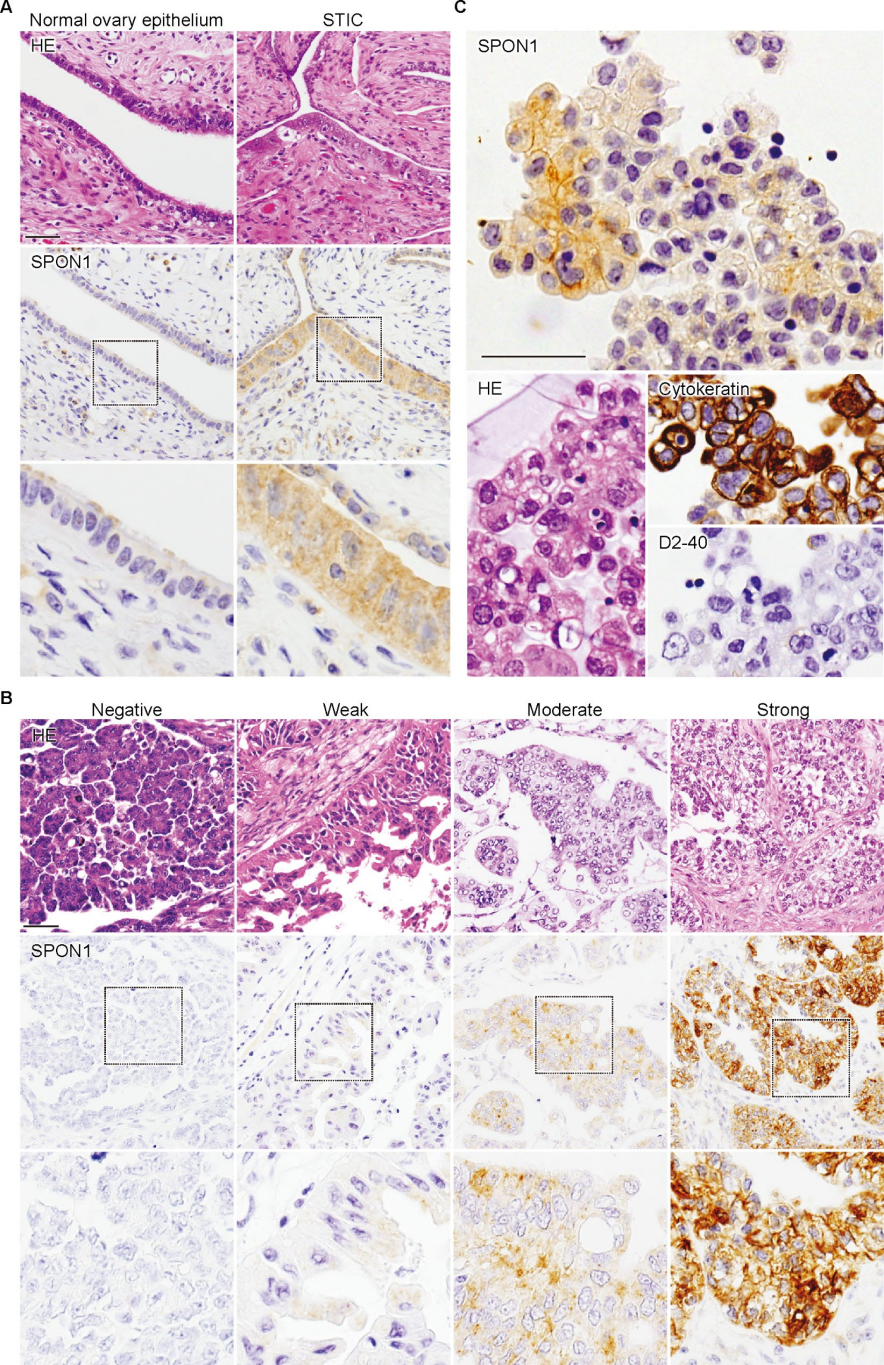
**Representative immunohistological images of SPON1 in normal and neoplastic ovarian tissues.** Normal ovary and STIC (serous tubal intraepithelial carcinoma) tissues (A), ovarian cancer tissues (B), and ascites-derived tumor cells from ovarian cancer patient (C) were immuno-stained with the anti-SPON1 mAb (clone #1). HE, hematoxylin-eosin. Scale bar, 100 μm

### Gene expression data

Comprehensive gene expression data for 33 types of cancer tissues by The Cancer Genome Atlas (TCGA) and 14 kinds of cancer cell lines by the Cancer Cell Line Encyclopedia (CCLE) were downloaded from cBioPortal (https://www.cbioportal.org/) and depmap portal (https://depmap.org/portal/), respectively. Comprehensive gene expression data from 31 types of heathy tissues were obtained from the Genotype-Tissue Expression (GTEx) portal (https://gtexportal.org/). Correlation between the expression of *SPON1* gene and the profile of tumor-infiltrating immune cells were analyzed by Tumor Immune Estimation Resource 2.0 database (TIMER 2.0; http://timer.cistrome.org/).

### Interactome analysis

The STRING database (https://string-db.org/) version 11.5 was used to analyze the protein-protein interaction (PPI) network. This database generates a network of PPI from high-throughput experimental data, literature and predictions based on genomic context analysis. The interactions in STRING are derived from five main sources: genomic context predictions, high-throughput lab experiments, co-expression, automated text mining and previous knowledge in databases [19].

### Statistical analysis

We used the fisher’s exact test to evaluate the relationship between SPON1 expression and various clinicopathological parameters. Survival analysis was performed using the Kaplan–Meier method, and differences between the groups were analyzed using the log-rank test. The logistic regression multivariable model was used to detect the independent predictors of survival. Two-tailed *P*-values < 0.05 were considered to indicate a statistically significant result. All statistical analyses were performed using GraphPad Prism 9 software (GraphPad Software, San Diego, USA) and StatFlex ver.7 (Artech, Osaka, Japan).

## Results

### Expression of SPON1 gene in a variety of normal tissues, cancer tissues, and cancer cell lines

Using TCGA, we first examined the expression of *SPON1* gene in various types of cancer. As shown in Figure S1A, *SPON1* mRNA appeared to be highly expressed in ovarian cancer but not in other malignant tumors. Additionally, the dataset from the GTEx revealed that *SPON1* transcripts were very weakly detected in a variety of normal adult organs of humans, which had expression levels that were extremely lower than those in cancer tissues (Figure S1B, C). On the other hand, upon searching the CCLE, *SPON1* mRNA was highly expressed not only in ovarian cancer cell lines but also in endometrial cancer cells (Figure S1D). Taken together with previous report [16], these results suggest that SPON1 represents a specific biomarker for ovarian cancer.

### Establishment of an anti-human SPON1 mAb

We subsequently attempted to generate, by the medial iliac lymph-node method [17], mAbs that specifically recognize SPON1 and can be applicable for immunohistochemistry of FFPE tissues. N-terminal 217–234 amino acid region was selected as the antigen, because it is completely conserved between humans and mice and is not found in other members of the thrombospondin family (Fig. 1A, B). Upon screening by ELISA, 32 of 145 hybridomas showed high reactivity to the immunized peptide. We next validated whether the candidate clones were able to detect positive signals by immunostaining of FFPE tissues using cell blocks of the SPON1-expressing 293T cells, and consequently picked up three clones (clones #1, #2, and #3) (Fig. 1C). Furthermore, on Western blot, clones #1 and #3 exhibited specific signals for SPON1 in *SPON1*-introducing 293T cells but not in control 293T cells, whereas clone #2 possessed no signals in these cells (Fig. 1D). The anti-SPON1 mAb (clone #1) also dose-dependently reacted to the SPON1 peptide by ELISA analysis (Fig. 1E). In addition, it recognized the endogenous SPON1 signals in three ovarian cancer cell lines by immunohistochemistry using their cell blocks (Fig. 1F). Therefore, we used clone #1 of anti-SPON1 mAb for further analysis.

**Fig. 3:**
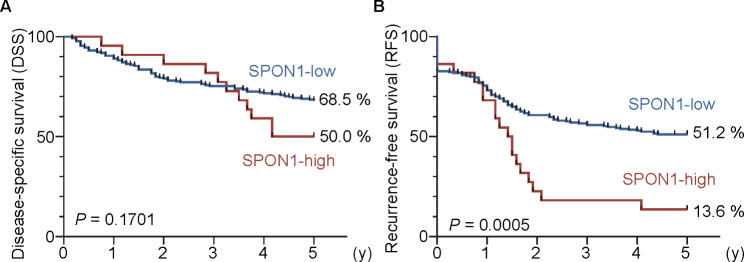
**High SPON1 expression is associated with poor outcomes in ovarian cancer patients.** (A) The disease-specific and (B) recurrence-free survival for the SPON1-low and SPON1-high expression groups in ovarian cancer subjects are indicated

### Expression of SPON1 in normal ovary, serous tubal intraepithelial carcinoma (SITC), and ovarian cancer tissues

Next, by immunohistochemical analysis using the established mAb, we evaluated the expression of SPON1 protein in normal ovary, serous tubal intraepithelial carcinoma (STIC), and ovarian cancer tissues, as well as in a range of normal adult tissues. No SPON1 signal was detectable in any tissues of the brain, heart, liver, kidney, lung, pancreas, testis, thyroid gland, or uterus (Figure S2). By contrast, the normal ovarian tissue was faintly positive for SPON1, whereas STIC showed the moderate cytoplasmic signals (Fig. 2A). SPON1 appeared to be observed in the cytoplasm of ovarian cancer tissues, but its signal intensity (SI) and percentage of positive cells (PP) were different among subjects (Fig. 2B). Furthermore, SPON1 was distributed not only in the cytoplasm but also along cell membranes in ascites-derived tumor cells (cytokeratin-positive and D2-40-negative) obtained from a patient with ovarian cancer (Fig. 2C).

**Table 1:** Relationship between SPON1 expression and clinicopathological factors in patients with ovarian cancer (n = 242)

Parameter		Total		Low SPON1 (N = 220)		High SPON1 (N = 22)			*P*-value
Age								0.258	
	< 60	149	(61.6)	138	(62.7)	11	(50.0)		
	≥ 60	93	(38.4)	82	(37.3)	11	(50.0)		
FIGO Stage								0.001	
	I/II	112	(46.3)	109	(49.5)	3	(13.6)		
	III/IV	130	(53.7)	111	(50.5)	19	(86.4)		
Histological type								0.058	
	High-grade serous	83	(34.3)	71	(32.3)	12	(54.5)		
	Non-high-grade serous	159	(65.7)	149	(67.7)	10	(45.5)		
	Low-grade serous	13	(5.4)	10	(4.5)	3	(13.6)		
	Mucinous/Endometrioid	78	(32.2)	75	(34.1)	3	(13.6)		
	Clear cell	56	(23.1)	54	(24.5)	2	(9.1)		
	Others/unknown	12	(5.0)	10	(4.5)	2	(9.1)		
T classification (pT)								0.011	
	1	95	(39.3)	92	(41.8)	3	(13.6)		
	2/3	147	(60.7)	128	(58.2)	19	(86.4)		
Lymph node metastasis								1.000	
	(–)	123	(50.8)	117	(53.2)	6	(27.3)		
	(+)	40	(16.5)	39	(17.7)	1	(4.5)		
	Unknown	79	(32.6)	64	(29.1)	15	(68.2)		
Distant metastasis								0.721	
	(–)	215	(88.8)	196	(89.1)	19	(86.4)		
	(+)	27	(11.2)	24	(10.9)	3	(13.6)		
Peritoneal dissemination								< 0.001	
	(–)	123	(50.8)	120	(54.5)	3	(13.6)		
	(+)	119	(49.2)	100	(45.5)	19	(86.4)		
Ascites cytology								0.418	
	(–)	70	(28.9)	65	(29.5)	5	(22.7)		
	(+)	85	(35.1)	75	(34.1)	10	(45.5)		
	Unknown	87	(36.0)	80	(36.4)	7	(31.8)		
Type of surgery								0.009	
	Complete/optimal	116	(47.9)	111	(50.5)	5	(22.7)		
	Suboptimal	60	(24.8)	50	(22.7)	10	(45.5)		
	Unknown	66	(27.3)	59	(26.8)	7	(31.8)		
CA-125 (U/mL)								0.085	
	< 35	30	(12.4)	30	(13.6)	0	(0.0)		
	≥ 35	204	(84.3)	183	(83.2)	21	(95.5)		
	Unknown	8	(3.3)	7	(3.2)	1	(4.5)		
Platinum sensitivity								0.601	
	Sensitive	160	(66.1)	144	(65.5)	16	(72.7)		
	Resistance	61	(25.2)	57	(25.9)	4	(18.2)		
	No chemotherapy	21	(8.7)	19	(8.6)	2	(9.1)		
Neoadjuvant chemotherapy								0.405	
	(–)	222	(91.7)	203	(92.3)	19	(86.4)		
	(+)	20	(8.3)	17	(7.7)	3	(13.6)		
Recurrence								< 0.001	
	(–)	152	(62.8)	147	(66.8)	5	(22.7)		
	(+)	90	(37.2)	73	(33.2)	17	(77.3)		

**Table 2:** Univariable analysis of recurrence-free survival in ovarian cancer patients

Variable		HR	95% CI	*P*-value
Age	≥ 60	1.337	0.878–2.036	0.175
FIGO Stage	III/IV	6.215	3.760–10.271	< 0.001
Histological type	HGSC	3.602	2.320–5.592	< 0.001
T classification (pT)	2/3	6.233	3.566–10.897	< 0.001
Lymph node metastasis	(+)	2.490	1.392–4.455	0.002
Distant metastasis	(+)	4.345	2.528–7.466	< 0.001
Peritoneal dissemination	(+)	5.945	3.719–9.502	< 0.001
Ascites cytoloy	(+)	3.731	2.032–6.848	< 0.001
Type of surgery	Suboptimal	5.927	3.667–9.581	0.015
CA-125 (U/mL)	≥ 35	2.812	1.226–6.447	0.014
Platinum sensitivity	Resistance	4.893	2.935–8.159	< 0.001
Neoadjuvant chemotherapy	(+)	3.261	1.883–5.649	< 0.001
SPON1	High	3.567	2.085–6.105	< 0.001

**Table 3:** Multivariable analysis of recurrence-free survival in ovarian cancer patients

Variable		HR	95% CI	*P*-value
FIGO Stage	III/IV	5.693	3.419–9.482	< 0.001
SPON1	High	2.250	1.303–3.884	0.003

Interestingly, SPON1 was also occasionally observed in stromal cells of SPON1-positive ovarian cancer tissues (Figure S3A). On the other hand, no SPON1 signal was detected in stromal cells of SPON1-negative ovarian cancer tissues. To gain an insight into the nature of SPON1-expressing stromal cells, we subsequently evaluated correlation between the expression of *SPON1* gene and the profile of tumor-infiltrating immune cells by the EPIC and MCP-counter methods of the TIMER 2.0 database. As shown in Figure S3B, *SPON1* gene expression was most significantly and positively connected with cancer-associated fibroblasts (CAFs) among tumor-infiltrating stromal and immune cells. In addition, SPON1-expressing stromal cells seemed to be also positive for αSMA (Figure S3A). Thus, these results suggested that SPON1 could be also expressed in αSMA-positive CAFs.

Based on semi-quantification using the immunoreactive score (IRS) [18], 22 of the 242 cases (9.1%) showed high SPON1 expression (score 3+). In the low-expression group, 64 (26.4%), 87 (36.0%), and 69 (28.5%) cases had scores 2+, 1+, and 0, respectively (Figure S4).

### High SPON1 expression is an independent poor prognostic marker for ovarian cancer

Kaplan − Meier plots revealed significant differences in RFS (*P* = 0.0005) but not in DSS (*P* = 0.1701) between the SPON1-high and -low groups (*P* = 0.1701) (Fig. 3). The 5-year RFS rates in the SPON1-low and SPON1-high groups were 51.2% and 13.6%, respectively.

Among the clinicopathological factors, high SPON1 expression was significantly associated with FIGO stages III/IV (*P* = 0.001), pT2/3 (*P* = 0.011), peritoneal dissemination (*P* < 0.001), type of surgery (*P* = 0.009), and recurrence (*P* < 0.001) (Table 1). In contrast, it did not relate to younger age (*P* = 0.258), histological type (high-grade serous vs. non-high-grade serous, *P* = 0.058), lymph node metastasis (*P* = 1.000), distant metastasis (*P* = 0.721), ascites cytology (*P* = 0.418), CA-125 (*P* = 0.085), platinum sensitivity (*P* = 0.601), or neoadjuvant chemotherapy (*P* = 0.405).

In the univariable analysis, FIGO stage III/IV (hazard ratio [HR] = 6.215, 95% confident interval [CI] 3.760–10.271, *P* < 0.001), high-grade serous carcinoma (HR = 3.602, 95% CI 2.320–5.592, *P* < 0.001), pT2/3 (HR = 6.233, 95% CI 3.566–10.897, *P* < 0.001), lymph node metastasis (HR = 2.490, 95% CI 1.392–4.455, *P* = 0.002), distant metastasis (HR = 4.345, 95% CI 2.528–7.466, *P* < 0.001), peritoneal dissemination (HR = 5.945, 95% CI 3.719–9.502, *P* < 0.001), ascites cytology (HR = 3.731, 95% CI 2.032–6.848, *P* < 0.001), type of surgery (HR = 5.927, 95% CI 3.667–9.581, *P* < 0.001), high levels of serum CA-125 (HR = 2.812, 95% CI 1.226–6.447, *P* = 0.014), platinum sensitivity (HR = 4.893, 95% CI 2.935–8.159, *P* < 0.001), neoadjuvant chemotherapy (HR = 3.261, 95% CI 1.883–5.649, *P* < 0.001), and high SPON1 expression (HR = 3.567, 95% CI 2.085–6.105, *P* < 0.001) exhibited significant prognostic variables for the RFS of ovarian cancer patients (Table 2).

Moreover, Cox multivariable analysis demonstrated that FIGO stage III/IV (HR = 5.693, 95% CI 3.419–9.482, *P* < 0.001) and high SPON1 (HR = 2.25, 95% CI 1.303–3.884, *P* = 0.0036) were independent prognostic factors for RFS of ovarian cancer (Table 3).

### Protein-protein interaction signature for SPON1

The STRING (Search tool for the retrieval of interacting genes/proteins) database displayed 11 nodes with 46 protein-protein interaction (PPI) networks with at least 2e-15 of enriched *P* value for SPON1 (Figure S5). The molecular function of this PPI network included integrin binding (false discovery rate [FDR] = 0.0035), heparin binding (FDR = 0.0035), and metalloendopeptidase activity (FDR = 0.0147). Furthermore, the STRING reactome pathway analysis indicated the involvement of SPON1 in post-translational modification (FDR = 7.45e-11) and extracellular matrix organization (FDR = 0.0038).

## Discussion

In the present study, we developed a specific anti-SPON1 mAb. The established mAb (clone #1) was applicable to ELISA, Western blot, and immunohistochemical analyses. Therefore, it should provide a powerful tool to verify the significance of SPON1 in diverse cells, tissues, and other samples.

Out of 242 cases of ovarian cancer tissues, we showed that SPON1 was weakly, moderately, and strongly expressed in 87 (36.0%), 64 (26.4%), and 22 (9.1%), respectively. The moderate SPON1 signals were also observed in STIC tissues examined. These SPON1-positive signals were distributed in the cytoplasm, whereas SPON1 was concentrated not only in the cytoplasm but also on cell membranes of ascites-derived ovarian cancer cells. On the other hand, the SPON1-immunoreactivity was very faint in the normal ovary tissue and was not detected in a variety of normal adult tissues, such as the brain, heart, liver, kidney, lung, pancreas, testis, thyroid, and uterus tissues. Thus, SPON1 could be promising as a specific protein biomarker for STIC and ovarian cancer.

We also concluded that high SPON1 expression represents an independent poor prognostic marker for ovarian cancer at time of initial surgery. The evidence for this conclusion was based on the following results: (1) the 5-year RFS rate in the SPON1-high group of ovarian cancer subjects (13.6%) was significantly lower than that in the SPON1-low group (51.2%); (2) the high SPON1 expression was significantly related with FIGO stages III/IV, pT2/3, peritoneal dissemination, type of surgery, and recurrence; (3) the univariable analysis revealed that high SPON1 expression was a significant prognostic factor for the RFS of ovarian cancer patients; (4) upon multivariable analysis, high SPON1 exhibited an independent prognostic marker for the RFS of ovarian cancer subjects.

Another issue that should be discussed is the validity of SPON1 as a potential serum biomarker for ovarian cancer. As describe above, SPON1 protein could be expressed in both STIC and ovarian cancer, while it was hardly detected in the normal tissues examined. In addition, *SPON1* mRNA was highly expressed in ovarian cancer tissues but not in other malignant tissues or various normal tissues, which was in good agreement with the results of a previous report [16]. Taken collectivity with the notion showing that SPON1 is a secreted protein, it is reasonable that SPON1 could be used as a serum biomarker for ovarian cancer and STIC. The utility of SPON1 alone or together with CA-125 [20–27] and/or Human epididymis protein 4 (HE4) [22, 28] should be determined in future experiments using serum samples from patients with ovarian cancer, other benign and malignant tumors, as well as samples from healthy individuals.

Two fusion genes *SPON1-NRG2α* and *SPON1-TRIM29*, though which is observed with extremely low frequency, have been recently identified in ovarian cancer [29, 30], suggesting the contribution of SPON1 in the pathogenesis of ovarian cancer. In addition, although SPON1 overexpression appeared to be basically restricted to ovarian cancer tissues, there are a few reports revealing that SPON1 promotes progression of other cancer types in vitro. For instance, it has been reported that SPON1 accelerates malignant behaviors in pancreatic cancer cells, such as cell proliferation, colony formation, and chemoresistance [31]. However, it is largely unknown how SPON1 contributes to advancing cancer progression. Interestingly, our interactome analysis indicated that SPON1 was expected to interact not only with APP but also with various proteins, including a disintegrin and metalloproteinase with thrombospondin motifs 1/5/13 (ADAMTS1/TS5/TS13) and ADAMTS-like protein. Since many of these proteins are identified as exosomal proteins by the Exocarta database (http://www.exocarta.org/) and are known to be involved in cancer progression [32], these interactions might play roles in ovarian cancer development. Additionally, SPON1-expressing CAFs might also influence on ovarian cancer advancement.

In summary, the present study highlighted that high SPON1 expression predicts poor prognosis of ovarian cancer. The frequency of high SPON1 expression in high-grade serous ovarian cancer was higher than that in non-high-grade serous ovarian cancer, although the difference was not significant (*P* = 0.058). The reason is unknown, but it is reasonable because STIC, a precursor legion for high-grade serous ovarian cancer, moderately expressed SPON1. In addition, the 5-year DSS in SPON1-high groups was low compared with that in SPON1-low groups, though the significant difference was not achieved (P = 0.1701). Thus, analysis of a large number of cases will be required to obtain more solid conclusions on the clinicopathological relevance of the high SPON1 expression in patients with ovarian cancer. Further studies are also needed to determine whether and how SPON1 is involved in ovarian cancer progression as well as whether SPON1 can be used as a serum biomarker for ovarian cancer.

## Electronic supplementary material

Below is the link to the electronic supplementary material.

Supplementary Material 1

## Data Availability

All data generated or analyzed during this study are included in this article and its online supplementary material. Further enquiries can be directed to the corresponding author.

## Abbreviations

ADAMTS: A disintegrin and metalloproteinase with thrombospondin motifs; amyloid-β precursor protein
APP: Amyloid-β precursor protein
CA-125: Cancer antigen-125
CAF: Cancer-associated fibroblast
CCLE: Cancer cell line encyclopedia
CI: Confident interval
DAB: 3’,3’-diaminobenzidine
EOC: Epithelial ovarian cancer
FDR: False discovery rate
FFPE: Formalin-fixed paraffin-embedded
FIGO: The international federation of gynecology and obstetrics
GTEx: Genotype-tissue-expression
HE4: Human epididymis protein 4
HR: Hazard ratio
IRS: Immunoreactive score
mAb: Monoclonal antibody
PPI: Protein-protein interaction
RFS: Recurrence-free survival
STIC: Serous tubal intraepithelial carcinoma
SPON1: Spondin-1
STRING: Search tool for the retrieval of interacting genes/proteins
TCGA: The cancer genome atlas
TIMER2.0: Tumor immune estimation resources 2.0
