# Supplementary material for: SPON1 is an independent prognostic biomarker for ovarian cancer
Source: J Ovarian Res. 2023 May 13;16:95. doi: 10.1186/s13048-023-01180-8 (PMC10182672; doi:10.1186/s13048-023-01180-8)
Supplement: Supplementary file 1 — Supplementary Material 1 [file 13048_2023_1180_MOESM1_ESM.docx]

Supplementary Information for

SPON1 is an independent prognostic biomarker for ovarian cancer

Ryoya Miyakawa, Makoto Kobayashi, Kotaro Sugimoto, Yuta Endo, Manabu Kojima,

Yasuyuki Kobayashi, Shigenori Furukawa, Tsuyoshi Honda, Takafumi Watanabe, Shigeyuki Asano,

Shu Soeda, Yuko Hashimoto, Keiya Fujimori, Hideki Chiba

Makoto Kobayashi, Hideki Chiba

Email: [makokoba@fmu.ac.jp](mailto:sugikota@fmu.ac.jp), hidchiba@fmu.ac.jp

This PDF file includes:

Figures S1 to S6


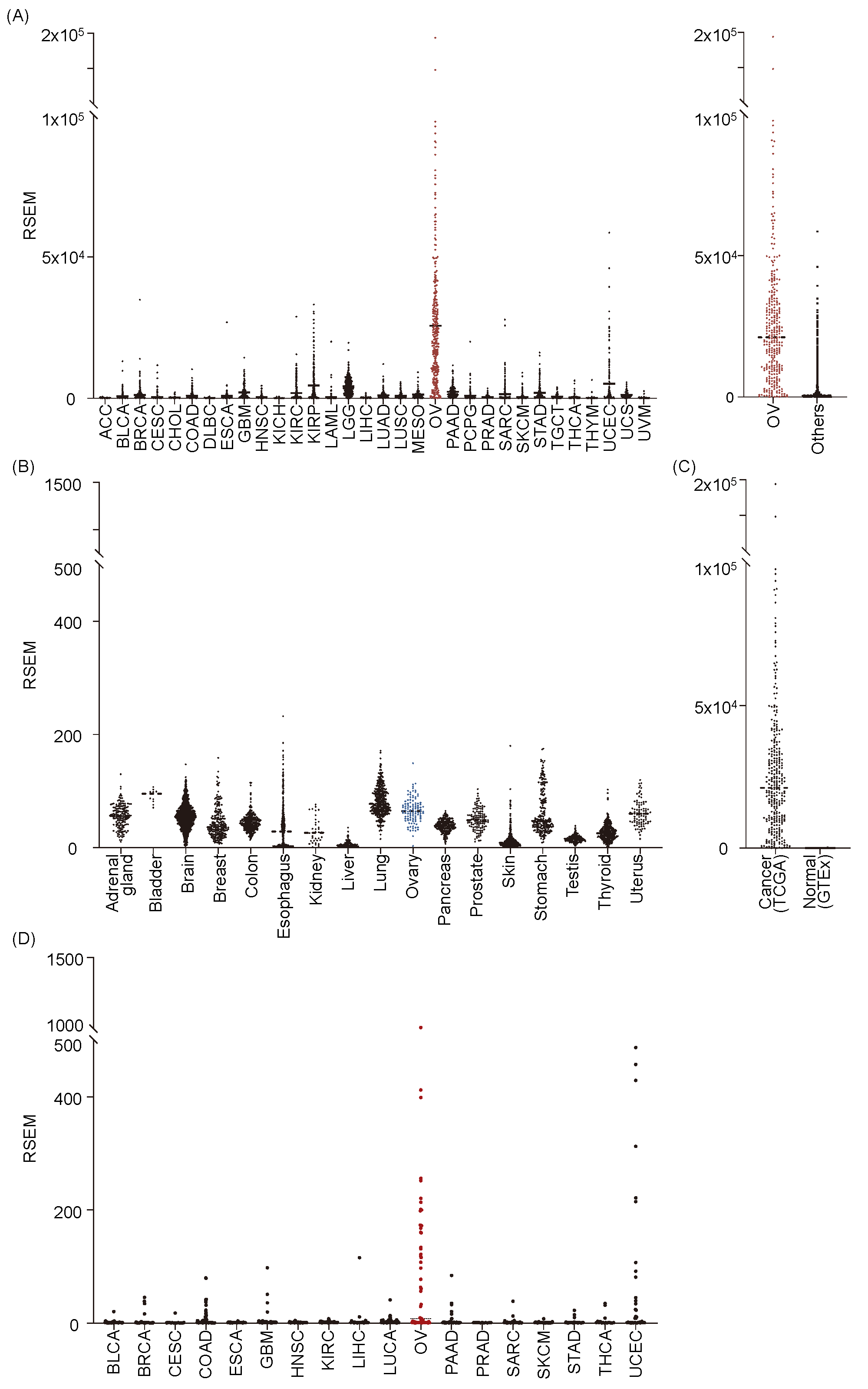


**Fig. S1. Expression of SPON1 gene in diverse normal tissues, cancer tissues, and cancer cell lines.** (A) The cancer genome atlas (TCGA) showing SPON1 mRNA levels in various cancer tissues. (B) The genotype-tissue expression (GTEx) revealing expression of SPON1 transcripts in the indicated normal tissues. (C) Expression of SPON1 gene in cancer and normal tissues. (D) The cancer cell line encyclopedia (CCLE) indicating expression levels of SPON1 mRNA in several cancer cell lines. Abbreviations are defined by the TCGA research network (https://gdc.cancer.gov/resources-tcga-users/tcga-code-tables/tcga-study-abbreviations). RSEM, RNA-seq by Expectation Maximization.

**
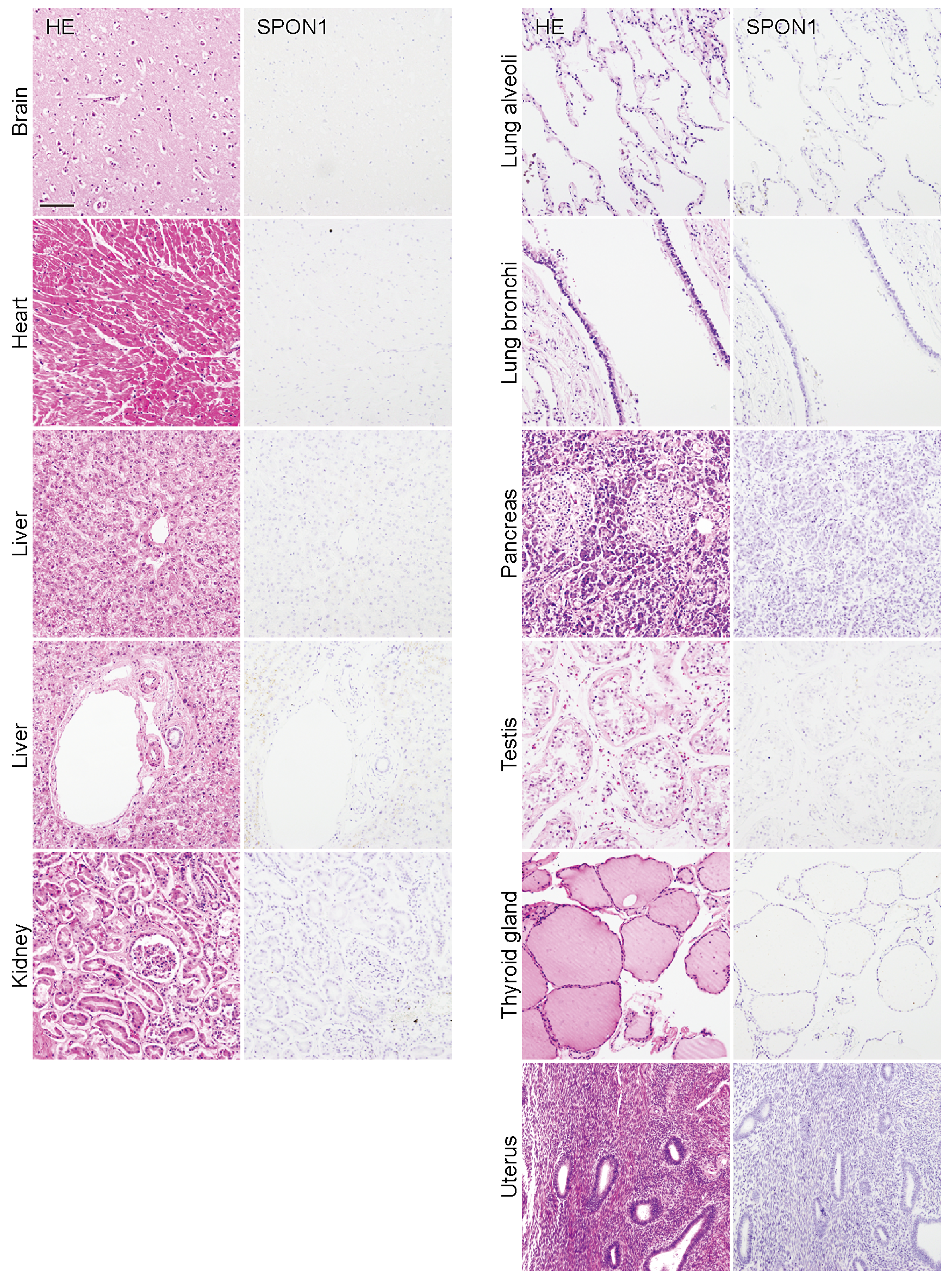
**

**Fig. S2. Absence of SPON1-positive signals in normal human tissues.** The indicated tissues were immunohistochemically stained with the anti-SPON1 mAb (clone #1). HE, hematoxylin-eosin. Scale bar, 100 µm.

**
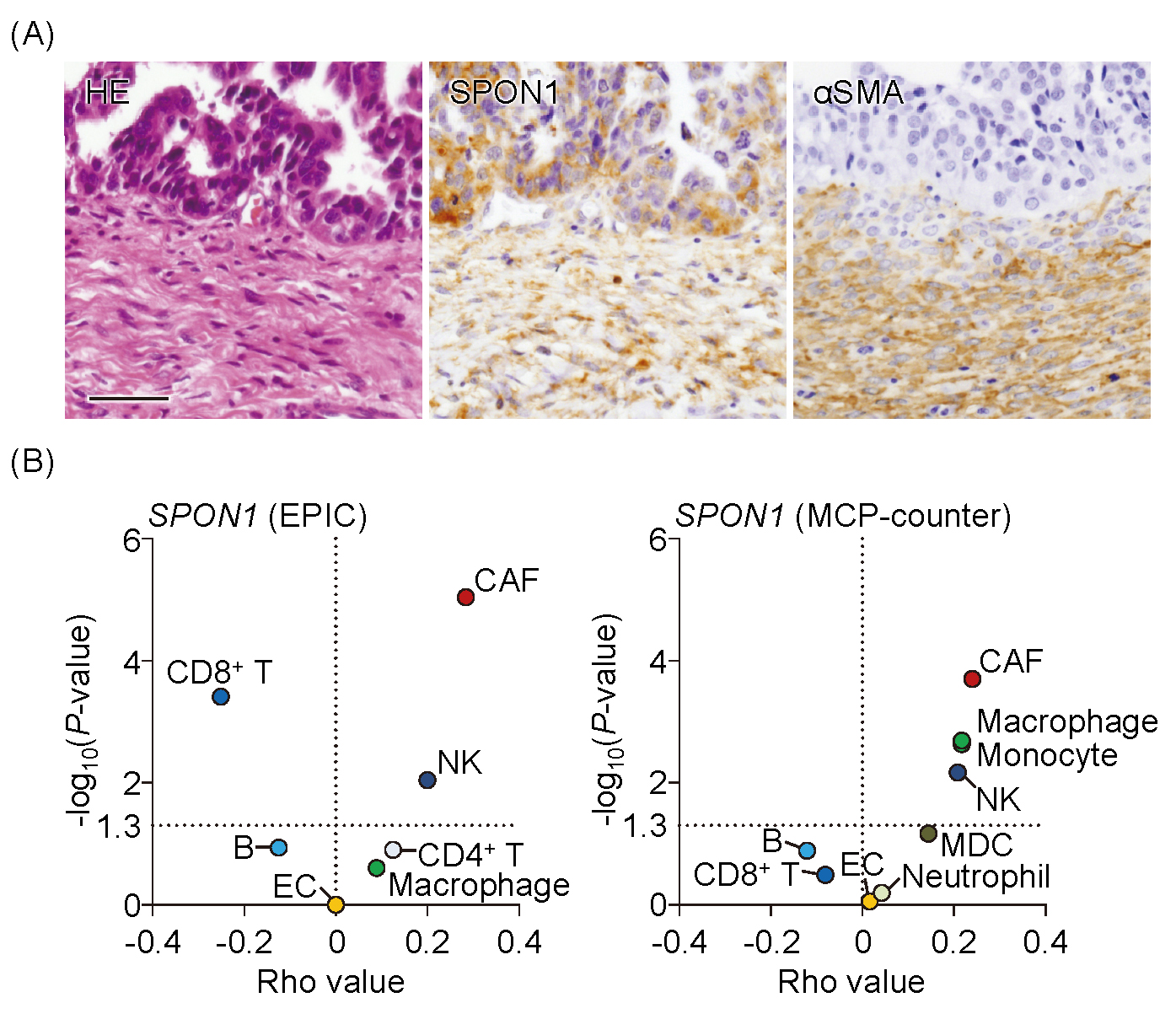
**

**Fig. S3. *SPON1* expression is strongly correlated with cancer-associated fibroblasts.** (A) Representative immunohistological images of SPON1 and αSMA in stromal cells of ovarian cancer tissues. HE, hematoxylin-eosin. Scale bar, 100 µm. (B) The Estimation of the Proportion of Immune and Cancer cells (EPIC) and the Microenvironment Cell Populations-counter (MCP-counter) in the TIMER 2.0 database revealing that *SPON1* gene expression is most significantly and positively connected with cancer-associated fibroblasts among tumor-infiltrating stromal and immune cells. B, B lymphocyte; CAF, cancer-associated fibroblast; EC, endothelial cell; MDC, myeloid dendritic cell; NK, natural killer cell; T, T lymphocyte.

**
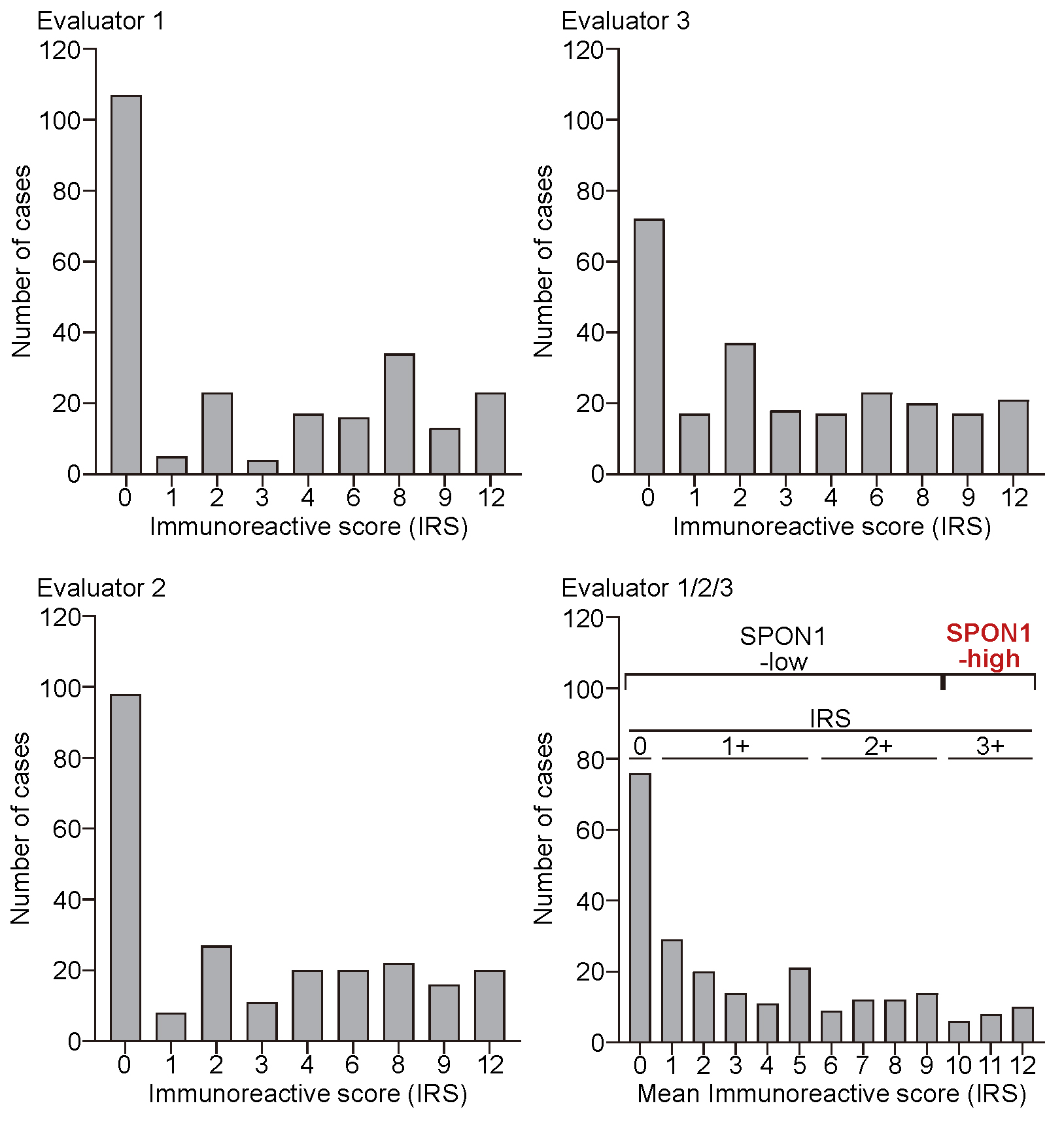
**

**Figure S4. Semi-quantification of the SPON1 signals in 242 cases of ovarian cancer tissues.** Two pathologists and a gynecologist quantified immunohistochemical staining using immunoreactive score (IRS), and their average score were calculated.

**
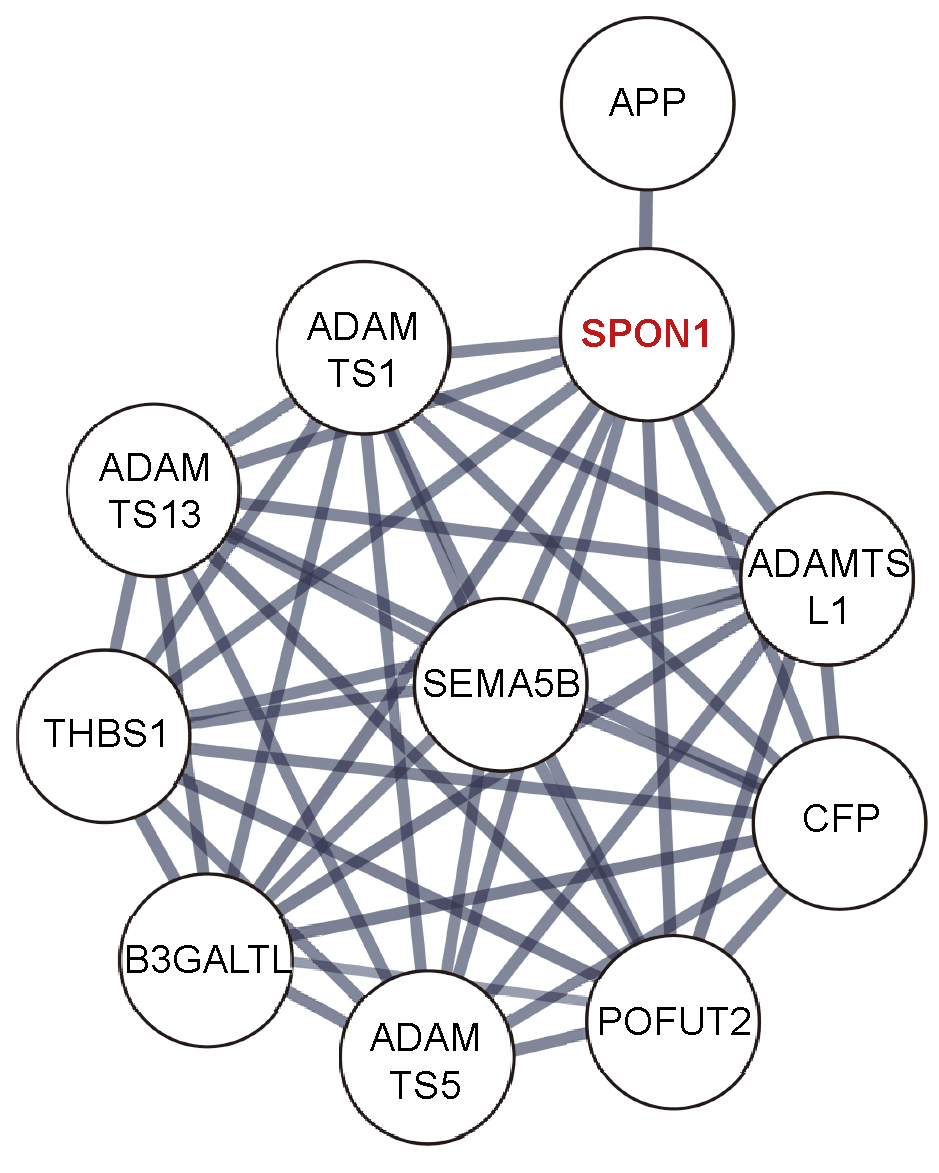
**

**Fig. S5.** **STRING database showing SPON1 protein-protein interaction signature.** The indicated proteins, except for APP (amyloid-β precursor protein), are predicted to be interact with SPON1 by computational analysis. The binding of SPON1 to APP is reported by Ho et al. 28. The lines reveal the expected interaction between each protein. ADAMTS1/TS5/TS13, A disintegrin and metalloproteinase with thrombospondin motifs 1/5/13; ADAMTSL1, ADAMTS-like protein 1; B3GALTL, UDP-glucose o-linked fucose beta-1,3-glucosyltransferase; CFP, Complement factor properdin; POFUT2, GDP-fucose protein O-fucosyltransferase 2; SEMA5B, Semaphorin-5B; THBS1, Thrombospondin-1.


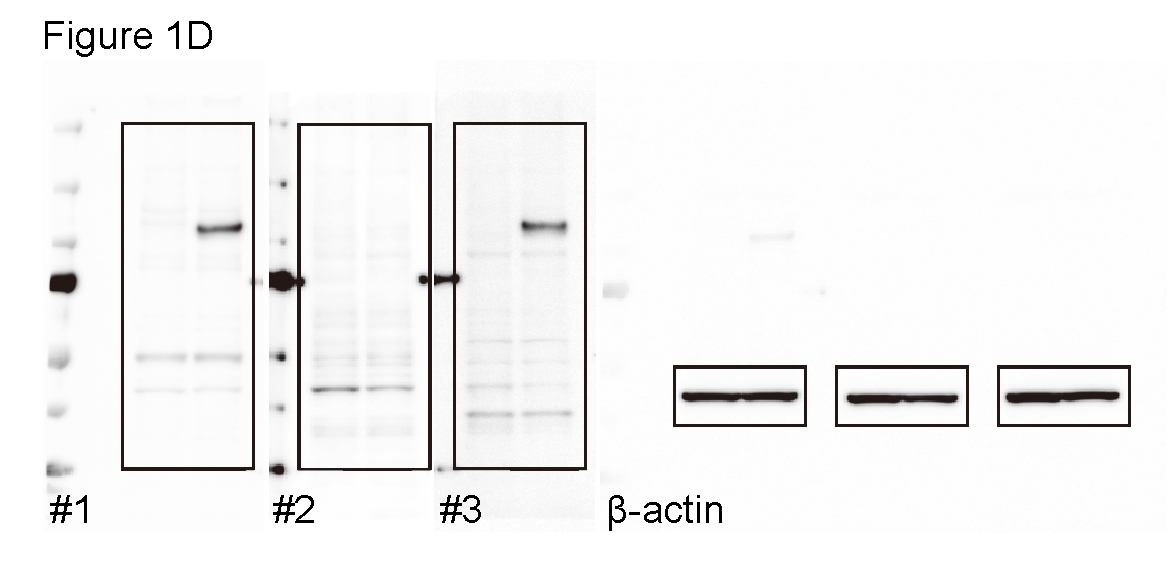


**Fig. S6. Uncropped images for the indicated western blot.**
